# BRIT1/MCPH1 Is Essential for Mitotic and Meiotic Recombination DNA Repair and Maintaining Genomic Stability in Mice

**DOI:** 10.1371/journal.pgen.1000826

**Published:** 2010-01-22

**Authors:** Yulong Liang, Hong Gao, Shiaw-Yih Lin, Guang Peng, Xingxu Huang, Pumin Zhang, John A. Goss, Francis C. Brunicardi, Asha S. Multani, Sandy Chang, Kaiyi Li

**Affiliations:** 1 The Michael E. DeBakey Department of Surgery, Baylor College of Medicine, Houston, Texas, United States of America; 2 Department of Systems Biology, M. D. Anderson Cancer Center, Houston, Texas, United States of America; 3 Department of Molecular Physiology and Biophysics, Baylor College of Medicine, Houston, Texas, United States of America; 4 Department of Molecular Genetics, M. D. Anderson Cancer Center, Houston, Texas, United States of America; The University of North Carolina at Chapel Hill, United States of America

## Abstract

BRIT1 protein (also known as MCPH1) contains 3 BRCT domains which are conserved in BRCA1, BRCA2, and other important molecules involved in DNA damage signaling, DNA repair, and tumor suppression. BRIT1 mutations or aberrant expression are found in primary microcephaly patients as well as in cancer patients. Recent *in vitro* studies suggest that BRIT1/MCPH1 functions as a novel key regulator in the DNA damage response pathways. To investigate its physiological role and dissect the underlying mechanisms, we generated *BRIT1* ^−/−^ mice and identified its essential roles in mitotic and meiotic recombination DNA repair and in maintaining genomic stability. Both *BRIT1* ^−/−^ mice and mouse embryonic fibroblasts (MEFs) were hypersensitive to γ-irradiation. *BRIT1* ^−/−^ MEFs and T lymphocytes exhibited severe chromatid breaks and reduced RAD51 foci formation after irradiation. Notably, *BRIT1* ^−/−^ mice were infertile and meiotic homologous recombination was impaired. *BRIT1*-deficient spermatocytes exhibited a failure of chromosomal synapsis, and meiosis was arrested at late zygotene of prophase I accompanied by apoptosis. In mutant spermatocytes, DNA double-strand breaks (DSBs) were formed, but localization of RAD51 or BRCA2 to meiotic chromosomes was severely impaired. In addition, we found that BRIT1 could bind to RAD51/BRCA2 complexes and that, in the absence of BRIT1, recruitment of RAD51 and BRCA2 to chromatin was reduced while their protein levels were not altered, indicating that BRIT1 is involved in mediating recruitment of RAD51/BRCA2 to the damage site. Collectively, our BRIT1-null mouse model demonstrates that BRIT1 is essential for maintaining genomic stability *in vivo* to protect the hosts from both programmed and irradiation-induced DNA damages, and its depletion causes a failure in both mitotic and meiotic recombination DNA repair via impairing RAD51/BRCA2's function and as a result leads to infertility and genomic instability in mice.

## Author Summary

The repair of DNA breaks in cells is critical for maintaining genomic integrity and suppressing tumor development. DNA breaks can arise from exogenous agents such as ionizing radiation (IR) or can form during the process of germ cell (sperm and egg) generation. BRIT1 protein (also known as MCPH1) is a recently identified DNA damage responding protein, and its mutations or reduced expression are found in primary microcephaly (small brain) patients, as well as in cancer patients. To investigate BRIT1's physiological functions and dissect the underlying molecular mechanism, we used a genetic approach (gene targeting technology) to delete BRIT1 gene in mice and generated a mouse model with BRIT1 deficiency (called BRIT1-knockout mice). Here, we showed that BRIT1 knockout mice are more sensitive to IR due to their inability to repair the IR-induced DNA breaks. These mice are also infertile, and their DNA repair during the process of germ cell generation was impaired substantially. Thus, in this study, we generated a novel mouse model (BRIT1 knockout mice) with striking phenotypes related to defective DNA repair and clearly demonstrated the essential role of BRIT1 in DNA repair at organism level.

## Introduction

The repair of DNA double-strand breaks (DSBs) is critical for maintaining genomic integrity [1],[2]. DSBs can arise from exogenous agents such as ionizing radiation (IR) [3] and endogenous factors such as stalled replication forks [4]. In addition, DSBs can form in a programmed manner during development including meiosis and immunoglobin rearrangements [5],[6]. During meiosis, DSBs are generated to initiate recombination between homologous chromosomes which leads to the reciprocal exchange of genetic materials between parental genomes. The inability for hosts to respond properly to the breaks or to repair them may trigger physiological defects such as infertility or cause genomic instability.

DNA damage response (DDR) pathways activated as a result of DSBs conceptually have three components, some with overlapping functions: sensors, signal transducers, and effectors [7],[8]. Damaged DNA is recognized by sensors; the signal is brought to transducers, which then in turn activate or inactivate the effectors that trigger cell cycle checkpoints, DNA repair or apoptosis. In response to DNA damage, many proteins involved in DDR pathway, including ATM [8], MDC1 [9], H2AX [10], NBS1 [11], 53BP1 [12],[13], RAD51 [14], BRCA1 [15], and BRCA2 [16], quickly accumulate to damage sites and form large nuclear aggregates that appear as IR-induced nuclear foci (IRIF) observed microscopically. A variety of evidence suggests that IRIF are required for precise and efficient DSB repair in the context of chromatin.

Recent studies suggest that BRIT1 (BRCT-repeat inhibitor of hTERT expression) is a key regulator for DNA damage response pathways [17],[18]. The sequence of *BRIT1* was derived from a hypothetical protein that was later matched to a putative disease gene called microcephalin (*MCPH1*), one of at least six loci implicated in the autosomal recessive disease primary microcephaly [19]. BRIT1 protein contains three BRCT (BRCA1 carboxyl terminal) domains, one in N-terminal and two in C-terminal. Many DDR and DNA repair proteins such as BRCA1, BRCA2, MDC1 and NBS1 contain BRCT domains, suggesting that BRIT1 may also play a role in DDR. In fact, knockdown of BRIT1 by specific siRNA in cells showed that BRIT1 was required for DNA damage-induced intra-S and G2/M checkpoints [17],[20],[21]. BRIT1 is also a chromatin-binding protein that forms IRIF, which co-localize with ATM, γ-H2AX, MDC1, NBS1 and 53BP1, and depletion of BRIT1 by its siRNA impairs the IRIF formation of ATM, MDC1, NBS1, and 53BP1 [17],[18], indicating that BRIT1 may exert a direct role in transmitting DNA damage signals. In addition, expression levels of *BRIT1* are decreased in several types of human cancer including breast and ovarian cancers [18], suggesting that *BRIT1* may function as a novel tumor suppressor gene. To better define its physiological role, here we generated *BRIT1* ^−/−^ mice and demonstrated *BRIT1*'s essential role in regulation of both programmed and IR-induced DNA damage responses.

## Results

### Generation of *BRIT1*-deficient mice

**Figure 1:**
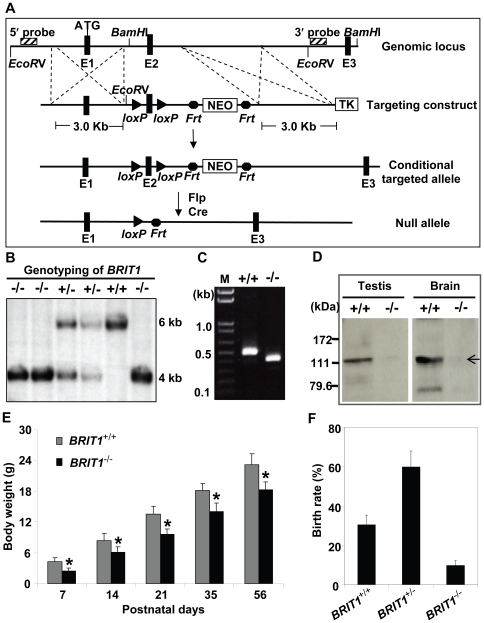
Generation and growth retardation of *BRIT1*-deficient mouse. (A) Schematic diagram of the *BRIT1* targeting strategy. The *BRIT1* targeting was described in detail in Materials and Methods. The positions of 5′ and 3′ flanking probes were shown as diagonally striped boxes. E1, E2, and E3 were the first three exons of *BRIT1*. (B) Genotyping of *BRIT1* ^+/+^, *BRIT1* ^+/−^ and *BRIT1* ^−/−^ mice. Genotyping was performed by Southern blot analyses. A 6 kb fragment was detected for WT and a 4 kb fragment was for the mutant mice. (C) Disruption of *BRIT1* transcript in *BRIT1* ^−/−^ mice. Total testes RNA from the indicated mice were extracted and subjected to RT-PCR. The products of RT-PCR were 514 bp (WT allele) and 422 bp (mutant allele). M, DNA marker. (D) Loss of BRIT1 protein in *BRIT1* ^−/−^ mice. Total proteins from the indicated tissues were extracted and subjected to Western blot using an anti-BRIT1 antibody. Arrow, the full-length BRIT1 protein. (E) *BRIT1* ^−/−^ mice were growth-retarded. Body weights of *BRIT1* ^+/+^ and *BRIT1* ^−/−^ littermates (n = 50 for each genotype in each age group) were measured at the postnatal day (P) 7, P14, P21, P35, and P56. *, *P*<0.05 compared with WT. (F) Lower birth rate of *BRIT1* ^−/−^ mice. Birth rates of *BRIT1* ^+/+^, *BRIT1* ^+/−^ and *BRIT1* ^−/−^ mice were calculated from the offspring of self-cross of *BRIT1* ^+/−^ mice (n = 400).

To characterize the physiological function of BRIT1, we generated *BRIT1* ^−/−^ mice by gene targeting (Figure 1A). The mice with germ-line transmission of the targeted conditional allele were crossed with *Flp* mice to eliminate the *Neo* cassette. To generate the global knockout mice, these mice were bred with transgenic mice carrying a *Cre* gene under the control of *β-actin* promoter to eventually generate *BRIT1* ^−/−^ mice, where exon 2 of *BRIT1* was deleted, leading to out of reading frame mutation of *BRIT1*.

We confirmed the loss of both *BRIT1* alleles in *BRIT1* ^−/−^ mice by Southern blot analysis (Figure 1B). RT-PCR with primers flanking exon 2 followed by DNA sequencing also revealed the disruption of *BRIT1* transcript in *BRIT1* ^−/−^ mice (Figure 1C). We also developed a rabbit antibody specifically against the C-terminal fragment of mouse BRIT1 and anti-BRIT1 Western blot confirmed the loss of BRIT1 in *BRIT1* ^−/−^ mice (Figure 1D).

*BRIT1* ^−/−^ mice were able to survive to adulthood, but they were growth-retarded (Figure 1E). The weight of *BRIT1* ^−/−^ mice at postnatal days 56 (P56) was only 80% compared to wild type (WT). In addition, birth rate of the *BRIT1* ^−/−^ mice was ∼10% among the offspring of self-cross of heterozygous mice (Figure 1F), which was much lower than normal Mendelian ratio (∼25%), suggesting that *BRIT1* deficiency may affect early development in mice.

### *BRIT1*-deficient mice and MEFs were hypersensitive to IR and defective in homologous recombination

**Figure 2:**
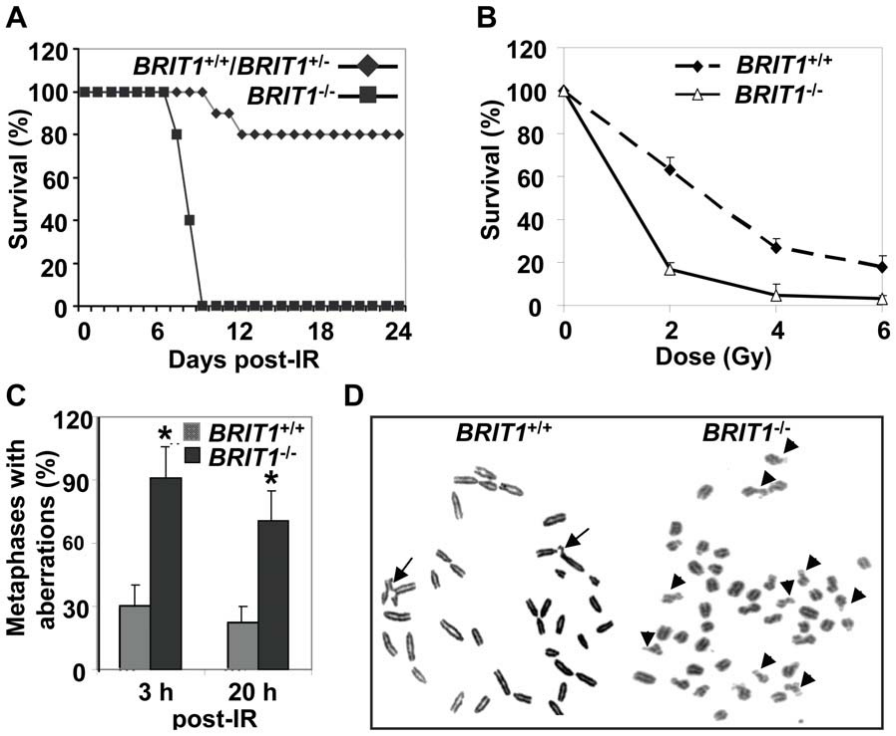
*BRIT1*-deficient mice and MEFs showed hypersensitive to γ-irradiation. (A) *BRIT1* ^−/−^ mice were hypersensitive to γ-irradiation (IR). *BRIT1* ^+/+^, *BRIT1* ^+/−^ and *BRIT1* ^−/−^ littermates (n = 30 for each genotype) were exposed to 7 Gy of whole-body IR, and were then monitored for 4 weeks. (B) *BRIT1* ^−/−^ MEFs were more sensitive to IR. Radiosensitivity of *BRIT1* ^+/+^ and *BRIT1* ^−/−^ MEFs was plotted as the fraction of surviving cells relative to unirradiated cells of the same genotype. (C,D) Enhanced chromosome aberrations in *BRIT1* ^−/−^ MEFs. *BRIT1* ^+/+^ and *BRIT1* ^−/−^ MEFs were γ-irradiated with 1 Gy, collected at 3 h or 20 h after IR, and subjected to metaphase spread assay. At least 30–35 metaphase spreads were analyzed for chromosome aberrations for each genotype. The main type of chromosome aberrations is chromatid breaks. Representative aberrations in *BRIT1* ^+/+^ and *BRIT1* ^−/−^ MEFs at 3 h post-IR were shown in (D). Arrows represent chromatid breaks.

One of the hallmarks of defective DNA damage response is increased radiation sensitivity. To assess whether the loss of BRIT1 expression renders mice hypersensitive to IR, we irradiated *BRIT1* ^+/+^, *BRIT1* ^+/−^ and *BRIT1* ^−/−^ mice with the dose of 7 Gy. All *BRIT1* ^−/−^ mice died within 9 days after irradiation, while 80% of *BRIT1* ^+/−^ or *BRIT1* ^+/+^ mice were still alive 4 weeks after irradiation (Figure 2A). We also examined *BRIT1* ^−/−^ MEFs' sensitivity to IR. Passage 2 primary MEFs were used to expose to different dosages of IR. The surviving cells were counted at the 6th day after IR. *BRIT1* ^−/−^ MEFs were more sensitive to IR as compared to *BRIT1* ^+/+^ cells (Figure 2B). Thus, both *BRIT1* ^−/−^ mice and the MEFs derived from those mice are more sensitive to irradiation.

To explore if genomic instability occur in *BRIT1* ^−/−^ cells, we performed metaphase spread assay to examine chromosome aberrations in both *BRIT1* ^+/+^ and *BRIT1* ^−/−^ MEFs shortly (3 h) and longer time (20 h) after IR. Metaphase spread showed that most of *BRIT1* ^−/−^ MEFs had DNA breaks, while only fewer of *BRIT1* ^+/+^ cells had breaks at as early as 3 h after IR (Figure 2C and 2D). Remarkably, at 20 h after IR, we still observed more chromosomal aberrations in *BRIT1* ^−/−^ than *BRIT1* ^+/+^ MEFs (Figure 2C). To assess if BRIT1 plays a role in regulating spontaneous DNA damage, we isolated T cells from both wild-type (WT) and mutant mice spleens and compared their genomic stability by metaphase spread, and found more chromosomal aberrations in the *BRIT1* ^−/−^ T cells than in WT (Table S1). While other types of chromosomal aberrations (e.g. translocations, polyploidy) also occurred in IR-treated *BRIT1* ^−/−^ MEFs or T lymphocytes, the majority aberration in these cells was chromatid breaks, a phenomenon associated with defective homologous recombination (HR) [22],[23]. Together with our previous studies [24],[25], these results indicate that loss of *BRIT1* leads to defective DNA repair in homologous recombination, and eventually cause genomic instability. Thus, BRIT1 is involved in regulating both spontaneous and IR-induced DNA damage responses.

### *BRIT1*-deficient mice were infertile and exhibited meiotic defects

**Figure 3:**
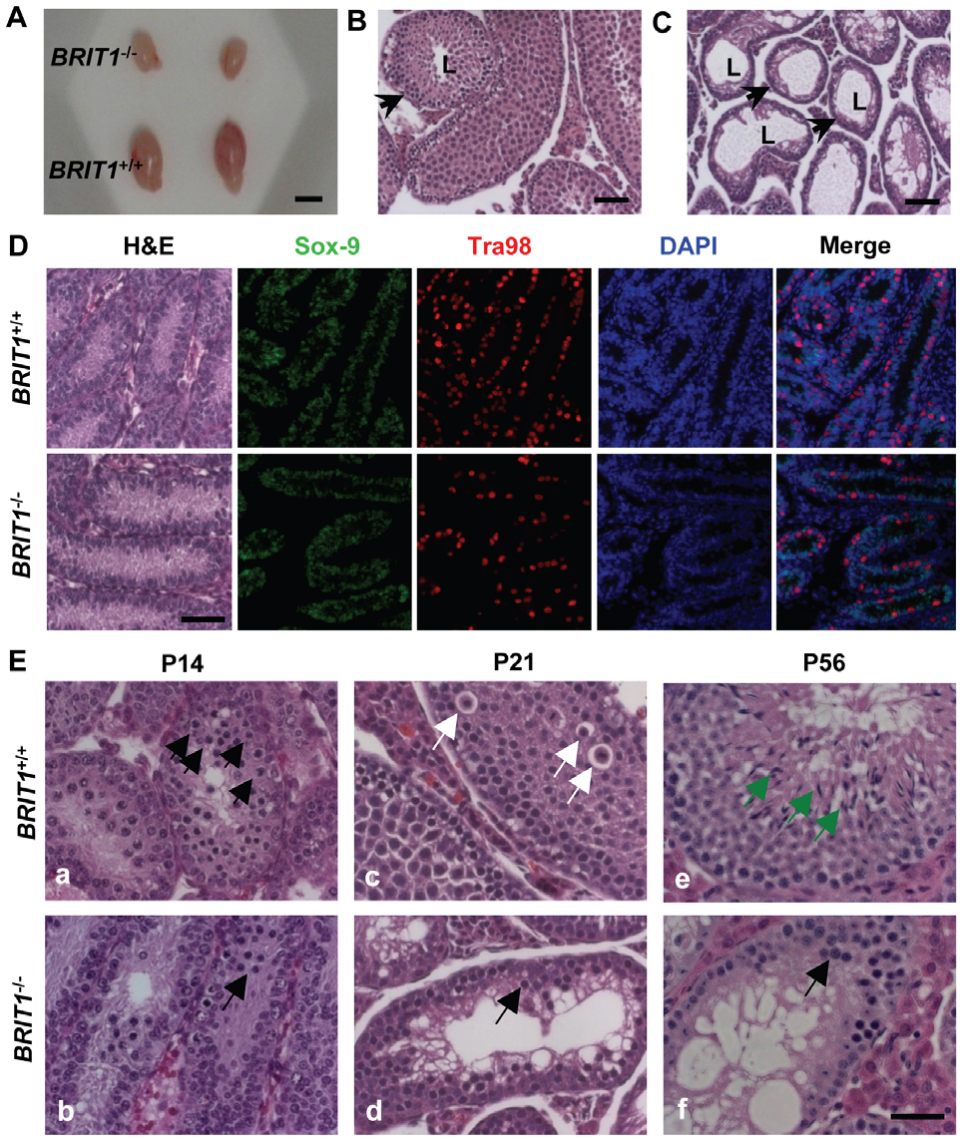
*BRIT1*-deficient male were infertile and exhibited meiotic defects. (A) Smaller testes in *BRIT1* ^−/−^ mice. Shown here are the testes from the indicated mice at P28. Scale bar, 5 mm. (B,C) Thinner seminiferous epithelia in *BRIT1* ^−/−^ mice. Testes sections from *BRIT1* ^+/+^ (B) and *BRIT1* ^−/−^ (C) mice at P28 were stained with haematoxylin-eosin (H&E). Arrows, the seminiferous tubules; L, lumen of the seminiferous tubules. Scale bar, 50 µm. (D) Mitosis was not defective in *BRIT1* ^−/−^ spermatogonia and Sertoli cells. Testes at P7 were either stained with H&E or double-stained with anti-Tra98 and anti-Sox9 antibodies using immunofluorescent staining. Scale bar, 50 µm. (E) Meiotic dysregulation in *BRIT1* ^−/−^ spermatocytes. H&E staining was performed in testis sections from *BRIT1* ^+/+^ and *BRIT1* ^−/−^ littermates at P14, P21, and P56. Black arrows: spermatocytes at leptotene/zygotene (a, b, d, f); white arrows: spermatocytes at diplotene (c); green arrows: elongate spermatids (e). Scale bar, 50 µm.

*BRIT1* ^−/−^ male mice failed to yield any pregnancies when crossed with the WT female mice, suggesting that *BRIT1* ^−/−^ mice are infertile. Consistently, we found that *BRIT1* ^−/−^ testes were much smaller than WT, especially in the mutant mice at the age of 3- week or older (Figure 3A and Figure S1). The testicular tubes in *BRIT1* ^−/−^ mice were significantly smaller and thinner than those in WT, and much fewer spermatocytes were produced in *BRIT1* ^−/−^ seminiferous tubules (Figure 3C, compared with Figure 3B), suggesting spermatogenesis is dysregulated due to loss of BRIT1. In addition, *BRIT1* ^−/−^ female mice are also infertile, and consistently, these mice harbored the smaller ovaries with none of ovarian follicles (Figure S2).

In the male mice, spermatogenesis is divided into several distinct stages: mitotic proliferation of spermatogonia, meiotic division of spermatocytes, and spermiogenesis of spermatids [26],[27]. To determine which stage of spermatogenesis is disrupted in *BRIT1* ^−/−^ mice, we collected the *BRIT1* ^+/+^ and *BRIT1* ^−/−^ testes at various developmental stages for histological analysis. At postnatal days 7 (P7), major cells were Sertoli or Sertoli-like cells (the supporting cells for testes) and spermatogonia, but no spermatocytes were found in either *BRIT1* ^+/+^ or *BRIT1* ^−/−^ testes (Figure 3D, H&E panel), consistent with the fact that spermatocytes do not form in mouse testes until around P10 [26],[27]. Testes sections at P7 were also double-immunostained with anti-Tra98 and anti-Sox9 antibodies to detect spermatogonia and its surrounding Sertoli cells, respectively. The number of both spermatogonia (Figure 3D, Tra98 panel) and Sertoli cells (Figure 3D, Sox9 panel) was comparable between *BRIT1* ^−/−^ and *BRIT1* ^+/+^ testes, indicating that *BRIT1* deficiency does not impair spermatogonia or Sertoli cell proliferation.

We next examined the development of spermatocytes using the mice at the age of 2- week or older. In testes at P14, P21 and P28, although spermatocytes had taken place in seminiferous tubules in both *BRIT1* ^−/−^ and *BRIT1* ^+/+^ testes, there were considerably fewer spermatocytes in *BRIT1* ^−/−^ as compared to *BRIT1* ^+/+^, especially after around P21 (panels c and d in Figure 3E). At P56, in addition to much smaller seminiferous tubules, remarkably fewer spermatocytes and no spermatids were found in *BRIT1* ^−/−^ testes (panels e and f in Figure 3E). In contrast to seminiferous tubules in WT with a full spectrum of spermatogenic cells (panels a, c and e in Figure 3E), *BRIT1* ^−/−^ tubules just contained two or three layers of darkly stained zygotene-like germ cells and exhibited a complete lack of pachytene spermatocytes and postmeiotic germ cells (panels b, d and f in Figure 3E), suggesting that meiosis in *BRIT1* ^−/−^ spermatocytes may arrest prior to the pachytene stage. Moreover, TUNEL assay revealed dramatically increased apoptosis in *BRIT1*-deficient tubules (Figure S1), suggesting degenerated spermatocytes in *BRIT1* ^−/−^ testes may be eliminated through apoptosis. Together, these data indicate spermatocyte meiosis is impaired due to loss of BRIT1, as a result, leads to the male infertility.

### *BRIT1*-deficient spermatocytes were arrested prior to the pachytene stage with aberrant chromosomal synapsis

**Figure 4:**
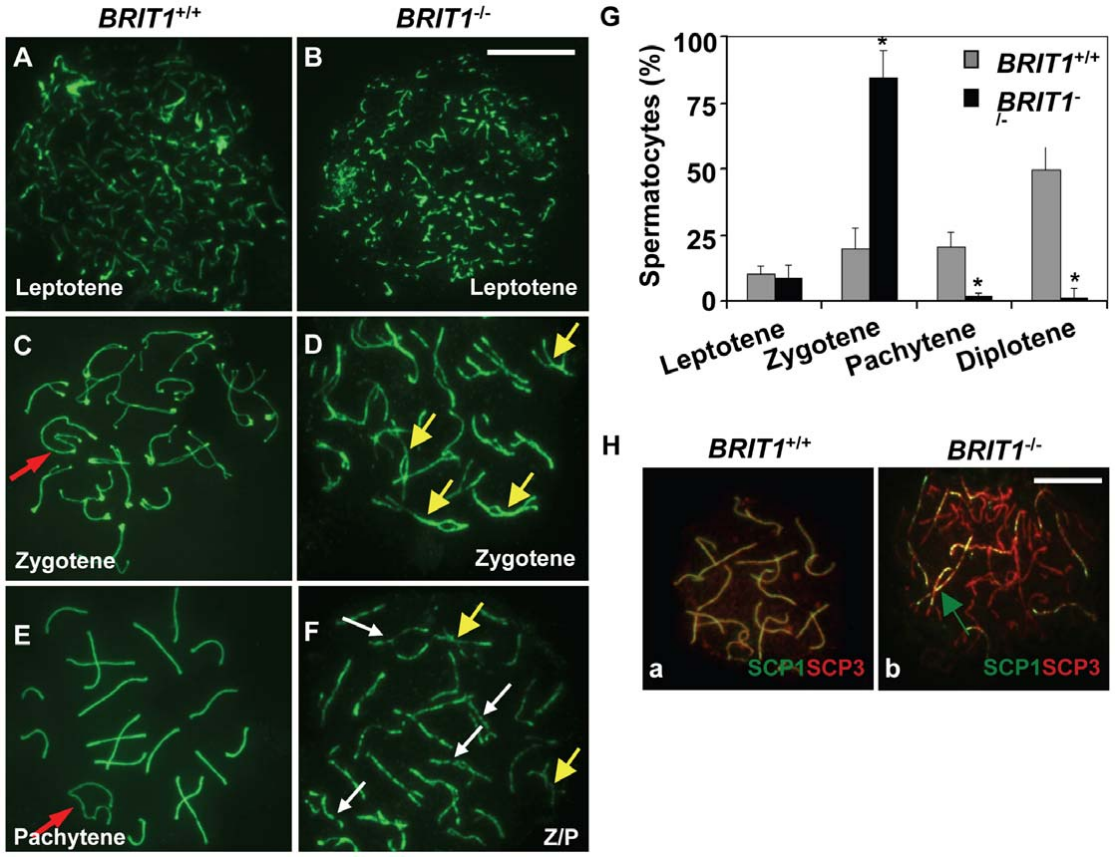
Meiosis in *BRIT1*-deficient spermatocytes was arrested prior to the pachytene stage with aberrant chromosomal synapsis. The staging of spermatocytes was examined in three pairs of WT and mutant mice by using anti-SCP3 immunostaining of the spermatocyte nuclei spread. (A,B) No defects in *BRIT1^−/−^* leptotene spermatocytes. The leptotene spermatocytes were detected in WT (A) and *BRIT1* ^−/−^ testes (B). Scale bar, 10 µm. (C,D) Aberrant bivalents in *BRIT1* ^−/−^ zygotene spermatocytes. Compared to the WT (C) with normal synapsis initiated typically at the distal ends of the acrocentric chromosomes, many mutant bivalents showed interstitial initiation of synapsis with asynapsis on either side of the contact (D). Red arrow, sex body; yellow arrows, interstitial synapsis. (E,F) *BRIT1* ^−/−^ spermatocytes were prone to be fragmented prior to the pachytene stage. Unlike WT with typical pachytene morphology (E), the mutant (F) was arrested prior to the pachytene stage or zygotene/pachytene transition stage and showed fragmented chromosome (white arrows). Red arrow, sex body; yellow arrows, interstitial synapsis; Z/P represents aberrant late zygotene/pachytene transition stage; white arrows, fragmented bivalents. (G) *BRIT1* ^−/−^ spermatocytes were dramatically arrested prior to the pachytene stage. Three to five hundred spermatocytes at meiosis I during the first wave were counted. Data are plotted as average percentage (mean±SD) determined from three pairs of mutant and WT mice. *, *P*≤0.01 (mutant versus WT). (H) Defective synapsis in *BRIT1^−/−^* spermatocytes. Synapsis here was determined by SCP1 staining. The complete bivalents were detected at zygotene/pachytene transition in WT spermatocytes (a). However, the mutant spermatocytes exhibited incomplete, dashed-line shape bivalents in many homologs during zygotene or zygotene/pachytene transition stage (b). Green arrow represents the incomplete, dashed-line shape synapsis (bivalents). Scale bar, 10 µm.

To determine the cause of meiotic arrest in *BRIT1* ^−/−^ spermatocytes, we sought to define the actual meiotic stage that was defected in the mutant by examining the assembly of the synaptonemal complex (SC). SC morphology in spermatocyte nuclei can be assessed by immunostaining of synpatonemal complex protein 3 (SCP3), an integral component of the axial/lateral elements in the SCs [28],[29]. SCP3-immunostaining of the spermatocyte nuclei spreads showed that all the substages of spermatocytes in meiotic prophase I were detected in WT mice such as leptotene (Figure 4A), zygotene (Figure 4C), pachytene (Figure 4E). In *BRIT1* ^−/−^ mice, spermatocytes at leptotene (Figure 4B) or zygotene (Figure 3D) stage were also detected. However, no typical pachytene chromosome morphology was detected though there were aberrant pachynema in *BRIT1* ^−/−^ spermatocytes (Figure 4F). In addition, there were much more zygotene spermatocytes found in the mutant testes as compared to those in WT (Figure 4G), indicating that loss of BRIT1 leads to meiotic arrest prior to the pachytene stage. Furthermore, in contrast to WT spermatocytes in which synapsis initiated typically at the distal ends (or in subtelomeric regions) of the acrocentric chromosomes [30] (Figure 4C), many mutant spermatocytes in mid- and late- zygotene were characteristic of interstitial initiation of synapsis with asynapsis on either side of the contact (Figure 4D and 4F). Also, the bivalents in the late zygotene or aberrant pachytene were prone to be fragmented (Figure 4F). To confirm the synapsis defects in *BRIT1* ^−/−^ spermatocytes, we performed anti-SCP1/SCP3 double-staining assay. SCP1 mediates synapsis via uniting homologous chromosomes during zygotene and pachytene stages. The accumulation of SCP1 on/around the axial/lateral elements of the whole SCs (indicated by SCP3) is a hallmark for complete synapsis [31]. In the mutant spermatocytes, SCP1/SCP3 double-staining assay showed incomplete, dashed-line like SCP1 pattern occurred in many individual homologs at the late zygotene or zygotene/pachytene transition stage (panel b in Figure 4H) while in WT, the intact accumulation of SCP1 protein around the whole SCs was detected (panel a in Figure 4H), revealing a defective synapsis existed in *BRIT1*-deficient spermatocytes. Collectively, these results indicate that meiosis in *BRIT1* ^−/−^ spermatocytes is arrested prior to the pachytene stage and BRIT1 is required for completing chromosomal synapsis during male meiosis.

### Recombination DNA repair was impaired in *BRIT1*-deficient spermatocytes

To further assess BRIT1's role in meiotic recombination, we examined BRIT1 expression pattern in seminiferous tubules as well as its foci formation in response to DSB during meiosis. We found that BRIT1 protein was expressed both in spermatogonia and spermatocytes (Figure S3A). Spermatocyte-chromosome spreading assay showed that BRIT1 foci were formed on the meiotic chromosomes during both leptotene and zygotene. In contrast, during pachytene where synapsis is completed, BRIT1 foci were not found on the chromosomes except in telomeres and non-synapsed sex body (Figure S3B). These data together indicate that BRIT1 is involved in repair of the DSBs occurring at leptotene and zygotene stages during meiotic recombination.

**Figure 5:**
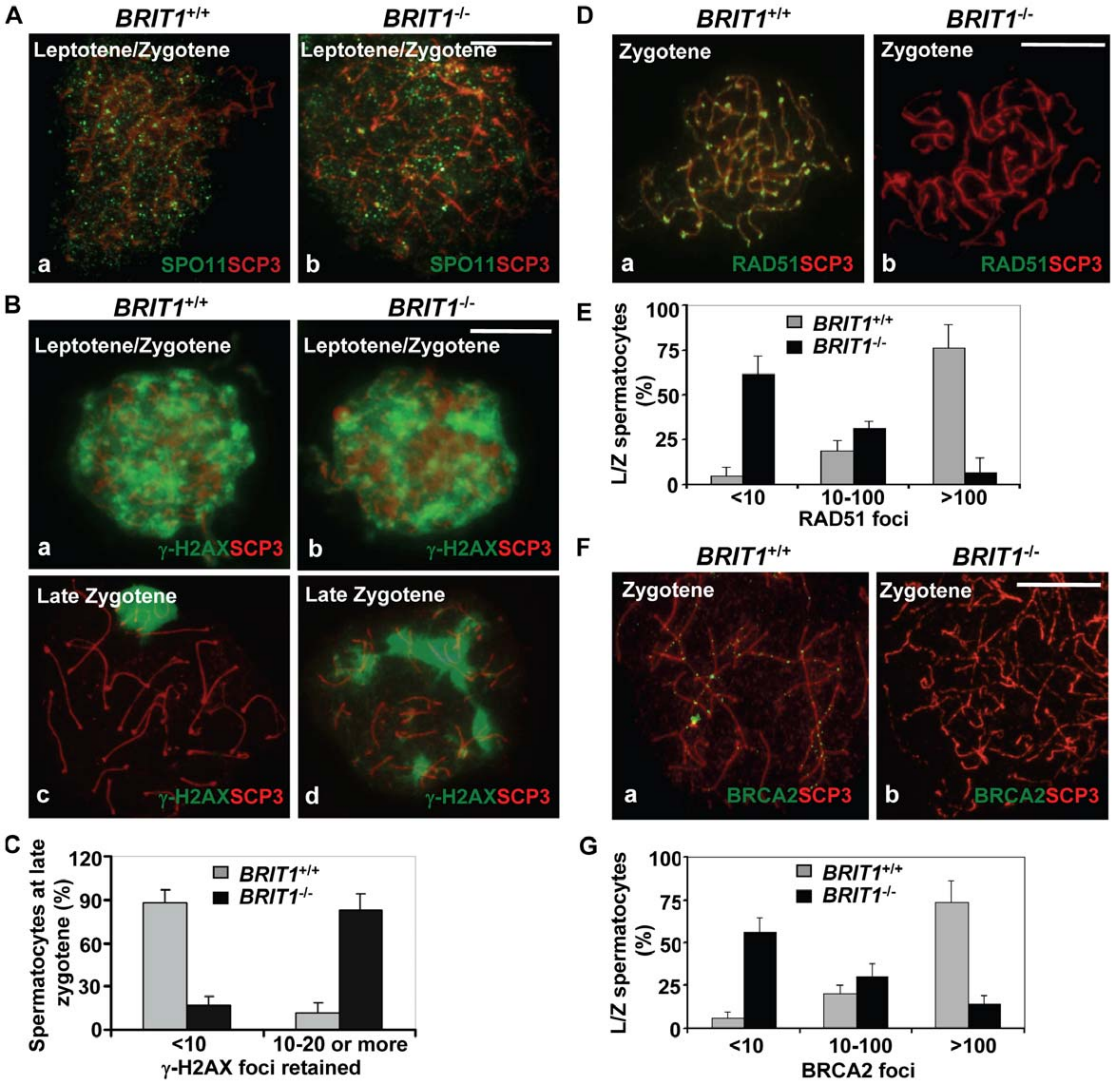
Recombination DNA repair was impaired in *BRIT1*-deficient spermatocytes. *BRIT1* ^+/+^ and *BRIT1* ^−/−^ spermatocytes were obtained from the indicated mice (P17.5–P22.5), and subjected for chromosome spreading and then double-stained immunofluorescently with anti-SCP3/anti-SPO11, anti-SCP3/anti-γ-H2AX, anti-SCP3/anti-RAD51, or anti-SCP3/anti-BRCA2 antibodies, respectively. Anti-SCP3 staining was used to determine the stage of each spermatocyte. Total around 400 chromosome spreads from three sets of mice were analyzed. Scale bar, 10 µm. (A) DNA double-strand breaks (DSBs) formation in meiosis is not affected by loss of Brit1. SPO11 foci had the similar pattern in WT and *BRIT1*-deficient spermatocytes. (B) γ-H2AX foci formation at leptotene/zygotene was comparable between WT and mutant spermatocytes. Both WT (a) and mutant (b) spermatocytes exhibited abundant γ-H2AX foci in response to DSBs at leptotene/zygotene stages, although γ-H2AX foci disappeared at the late zygotene/pachytene stage except in the XY body in WT (c), and they were sustained on asynapsed autosomal homologs in the mutant spermatocytes at late zygotene (d). (C) Retained γ-H2AX foci during late zygotene spermatocyte in *BRIT1*-deficient spermatocytes. Value shown here represents the mean±SD from ∼50 late zygotene spermatocytes for each genotype. (D) RAD51 foci were dramatically decreased in zygotene stage of *BRIT1* ^−/−^ spermatocytes. RAD51 foci were formed intensively on zygotene homologs in WT (a) while few foci were detected in the *BRIT1* ^−/−^ zygotene (b). (E) Remarkable reduction in the number of RAD51 foci per leptotene/zygotene spermatocyte in *BRIT1*-deficient spermatocytes. Value shown here represents the mean ± SD from ∼50 leptotene/zygotene spermatocytes for each genotype. (F) BRCA2 foci formation was disrupted in *BRIT1* ^−/−^ spermatocytes. BRCA2 foci were formed intensively on bivalents at zygotene in WT (a) but not in the *BRIT1* ^−/−^ zygotene (b). (G) Substantial reduction in the number of BRCA2 foci per leptotene/zygotene spermatocyte in *BRIT1*-deficient spermatocytes. Value shown here represents the mean ± SD from ∼50 leptotene/zygotene spermatocytes for each genotype.

To investigate the mechanism mediating the meiotic defect due to loss of BRIT1, we examined if DSB formation and meiotic recombination repair was impaired. During meiosis, DSBs are introduced by SPO11 at leptotene for initiating meiotic recombination in spermatocytes [32], and γ-H2AX is an important protein involved in recognition and signaling of the DSBs [33],[34]. In mice, ∼300 DSBs are generated by SPO11 at leptotene/zygotene in each nucleus [35]. As shown in the Figure 5A, SPO11 foci had the similar pattern in WT and mutant spermatocytes, suggesting DSBs were normally formed in *BRIT1*-deficient testes. In response to the DSBs, γ-H2AX foci responded equally well and appeared at the damage sites during the leptotene stage of both the WT (panel a in Figure 5B) and the *BRIT1* mutant (panel b in Figure 5B) testes with the same staining pattern and comparable intensity. In consistent with previous report [36], at late zygotene/pachytene stage of WT spermatocytes, γ-H2AX staining disappeared from synapsed autosomal chromosomes though it still resided in the largely asynapsed sex chromosomes of the XY body (panel c in Figure 5B). However, γ-H2AX staining in the *BRIT1*-mutant spermatocytes was sustained in asynapsed autosomal homologs at late zygotene (Figure 5B and 5C). Thus, these data suggests that DSBs are normally formed, while DSBs cannot be properly repaired without functional BRIT1.

We next analyzed the homologous recombination process to address why DSBs are not repaired in *BRIT1* ^−/−^ spermatocytes. RAD51 is the homolog of *E. coli* RecA which binds to DSBs and plays a critical role in both mitotic and meiotic recombination DNA repair [37],[38]. In response to DNA damages, BRCA2 can facilitate RAD51's loading to the damages sites via their physical interaction and these two proteins form nuclear foci at the DSB sites and execute the DNA repair process [16]. We examined RAD51 foci formation on meiotic chromosomes using by immunostaining of chromosomes from spermatocytes. Interestingly, in *BRIT1* ^−/−^ leptotene/zygotene spermatocytes, the number of RAD51 foci was greatly decreased as compared to the number in WT (Figure 5D and 5E). We found that 80% of leptotene/zygotene spermatocytes in WT contained ∼100–250 RAD51 foci (Figure 5D and 5E). In contrast, 60% of the mutant spermatocytes at these stages exhibited no or only a few RAD51 foci (Figure 5D and 5E). In addition, RAD51 protein level was not changed in *BRIT1* ^−/−^ testes (Figure S4). Thus, reduction of RAD51 foci on chromosomes in *BRIT1*-deficient testes might be attributed to decreased localization of RAD51 onto the DSB sites. Furthermore, we found that BRCA2 foci formation in spermatocytes was also disrupted due to *BRIT1* deficiency (Figure 5F and 5G). Collectively, these data indicate that depletion of BRIT1 disrupts meiotic recombination repair through abolishing the recruiting and the function of RAD51/BRCA2, and as a result leads to the catastrophic meiosis failure in *BRIT1* ^−/−^ spermatocytes.

### BRIT1 is required for recruitment of RAD51/BRCA2 to the IR-induced DNA damage sites

**Figure 6:**
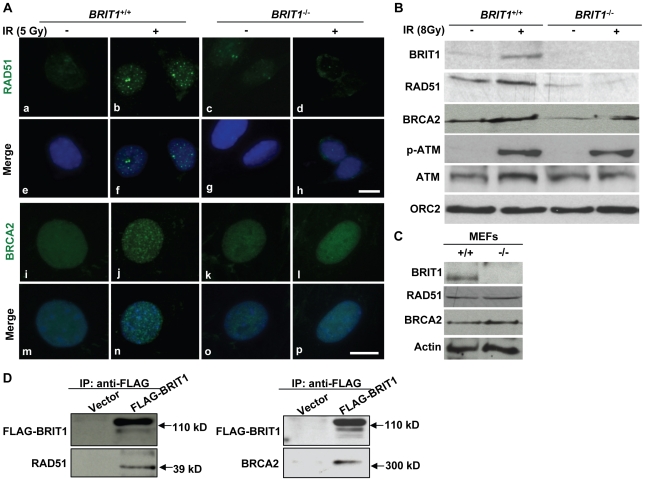
BRIT1 is required for recruitment of RAD51/BRCA2 to the IR-induced DNA damage sites. (A) RAD51/BRCA2 foci formation was inhibited in IR-treated *BRIT1* ^−/−^ MEFs. The WT and mutant MEFs on the coverslips were treated with or without ionizing radiation (5 Gy), then subjected to immunofluorescent staining 30 min later. IR-induced RAD51 foci were diminished in *BRIT1* ^−/−^ MEFs (d) compared to those in WT (b). Similarly, compared to WT (j), BRCA2 foci in mutant MEFs (l) were barely formed in response to IR. Scale bar, 10 µm. (B) Chromatin-bound RAD51 and BRCA2 were dramatically reduced in IR-treated *BRIT1* ^−/−^ MEFs. MEFs were treated with or without ionizing radiation (8Gy) and collected 1 h later. Chromatin pellets were then subjected to chromatin isolation and Western blot analysis, and probed with antibodies against BRIT1, RAD51, BRCA2, p-ATM, ATM or ORC2, respectively. ORC2 was used as a loading control. Chromatin-bound p-ATM or ATM remained the same between the WT and the mutant cells. However, there was much fewer RAD51 or BRCA2 bound to chromatin in the mutant MEFs as compared to the WT. p-ATM: phosphorylated ATM. (C) RAD51 or BRCA2 protein expression was not altered due to loss of BRIT1. Total protein lysates from indicated MEFs were used to detect the protein levels of RAD51/BRCA2. RAD51/BRCA2 expression was comparable between the WT and mutant MEFs. (D) BRIT1 physically associated with RAD51/BRCA2. The immortalized *BRIT1* ^+/+^ MEFs were transfected with vector or FLAG-BRIT1 and the cell lysates were collected 1 h after irradiation (8Gy), subjected to anti-FLAG immunoprecipitation assay, separated by SDS-PAGE, and blotted with anti-FLAG, anti-RAD51, or anti-BRCA2 antibodies, respectively.

To further explore how BRIT1 regulates DNA repair, we assessed the ability of RAD51/BRCA2 to form nuclear foci and their chromatin association in *BRIT1* ^−/−^ MEFs. We first analyzed the IR-induced nuclear foci (IRIF) formation of RAD51 and BRCA2 in *BRIT1* ^+/+^ and *BRIT1* ^−/−^ MEFs. As shown in Figure 6A, we observed typical RAD51 and BRCA2 foci in the *BRIT1* ^+/+^ MEFs, whereas only diffused pattern of RAD51 and BRCA2 were seen in the *BRIT1* ^−/−^ MEFs, indicating the IRIF formation of RAD51 and BRCA2 was abolished in the BRIT1 null background. In addition, in comparison to WT, the amount of chromatin-bound RAD51 was markedly reduced with or without IR treatment, indicating the requirement of BRIT1 for the basal and IR-induced Rad51 chromatin association. The basal chromatin-binding of BRCA2 was also significantly reduced in the BRIT1 null background while the IR-induced BRCA2 association to the chromatin was modest reduced (Figure 6B). The protein levels of RAD51 and BRCA2 were not altered due to lack of BRIT1 (Figure 6C). These data indicate that BRIT1 is required for increased basal chromatin affinity and the physical assembly of RAD51 and BRCA2 to the DNA damage loci though there is different degree of BRIT1 dependency in terms of IR-induced chromatin binding between RAD51 and BRCA2. Notably, we found that BRIT1 physically associated with RAD51 and BRCA2, suggesting BRIT1 being directly involved in DNA repair (Figure 6D). During the course of our study, a very recent report also shows that BRIT1 binds to BRCA2/RAD51 complex and disruption of the interaction between BRIT1 and BRCA2 leads to substantially reduced BRCA2/RAD51 at the DNA repair sites [39]. Altogether, these data indicate that BRIT1 may be directly involved in DNA repair via mediating RAD51/BRCA2's recruitment to the damage sites.

## Discussion

In this report, we generate a *BRIT1* knockout (*BRIT1* ^−/−^) mouse model and clearly demonstrate that BRIT1 is crucial for maintaining genomic stability *in vivo* to protect the hosts from both programmed and irradiation-induced DNA damages. Our studies on BRIT1 null mice also provide convincing evidence to identify a novel and important function of BRIT1 in meiotic recombination DNA repair. *BRIT1* ^−/−^ mice showed significant genomic instability, exemplified by chromosomal aberrations in T lymphocytes and MEFs. In addition, *BRIT1* ^−/−^ mice exhibited growth retardation, male infertility, and increased radiation sensitivity. These phenotypes are virtually identical to those of *ATM* ^−/−^, *MDC1* ^−/−^ and *H2AX* ^−/−^ mice [40]–[42], suggesting that these molecules integrate closely in the DDR pathway.

Importantly, this is the first reported evidence demonstrating that BRIT1 indeed plays a crucial physiological role in programmed DNA damage response and HR DNA repair *in vivo*. Our data presented here clearly reveal that BRIT1 is essential for meiotic recombination DNA repair in spermatocytes. We first demonstrated that the male infertility in null mice was caused by catastrophic meiosis failure in spermatocytes and accordingly, no spermatids could be generated (Figure 3E). We also showed that *BRIT1* ^−/−^ spermatocytes exhibited aberrant chromosomal synapsis, and meiosis was arrested before or at the transition of zygotene to pachytene of meiotic prophase I (Figure 4). In addition, we found that localization of RAD51/BRCA2 to meiotic chromosomes was severely impaired in the mutant spermatocytes while their protein levels were not altered due to loss of BRIT1 (Figure 5 and Figure S4). Thus, these data together indicate that the DSB generated by SPO11 at leptotene can not be repaired properly and as a result, leads to aberrant chromosomal synapsis and meiosis arrest at late zygotene stage. Interestingly, unlike MDC1 or H2AX whose deficiency only leads to male infertility [41],[42], loss of BRIT1 also lead to female infertility with much smaller ovary (Figure S2), suggesting that BRIT1 may also function as a key regulator in oocyte meiotic recombination.

During meiosis, DSB is generated by SPO11 that leads to the initiation of meiotic recombination (HR DNA repair) [32]. In the null mice, *BRIT1* deficiency only affected foci formation of RAD51/BRCA2, but not those of SPO11 and γ-H2AX, at leptotene/zygotene stages, Thus, our data support a model in which BRIT1 functions downstream of the SPO11-mediated DSB formation but upstream of RAD51/BRCA2-midiated DSB repair during meiotic recombination. In addition to meiosis, we also found that in response to DSBs induced by IR, the association of RAD51/BRCA2 to chromatin and their foci formation was impaired in MEFs with BRIT1 deficiency while their protein levels were not altered (Figure 6). All these *in vivo* and *in vitro* studies together therefore demonstrate that Brit1 is critical for DNA repair during both meiosis and mitosis. The impaired RAD51 foci formation in both *BRIT1* ^−/−^ MEFs and spermatocytes is not due to the changes of RAD51 protein levels (Figure 5 and Figure 6). Although BRIT1 has been reported to regulate RAD51 expression [43], we observed no altered RAD51 protein levels in either *BRIT1* ^−/−^ MEFs or the *BRIT1* ^−/−^ mouse testes which is consistent with our previous studies in BRIT1 knockdown human cells [25].

The mechanisms mediating BRIT1's function on DNA repair may be through multiple levels. Firstly, BRIT1 can be indirectly involved in DNA repair process via regulation of chromatin structure. We recently found that BRIT1 was associated with Condensin II, which modulates BRIT1-mediated HR repair [24]. In addition, we demonstrated BRIT1 being involved in chromatin remodeling via interacting with SWI/SNF, and this interaction relaxes the chromatin structure and increases the access of the repair proteins, including RAD51, to the DNA damage sites [25]. Indeed, this chromatin remodeling function of BRIT1 may also contribute to the increased accessibility of many other DNA damage responsors, such as ATM, ATR, NBS1, MDC1, 53BP1, RPA as we previously observed [18]. In addition to the generally increased affinity of DNA damage responsors and DNA repair proteins to chromatin, BRIT1 may further directly participate into DNA repair via interacting and recruiting RAD51/BRCA2 complex to the damage sites. Here, we observed that BRIT1 can physically associate with RAD51 or BRCA2 and in the absence of BRIT1, recruitment of RAD51 and BRCA2 to chromatin was remarkably reduced while their protein levels were not altered, suggesting that BRIT1 being directly involved in DNA repair (Figure 6). Consistently, in the course of our study, a very recent report also shows that BRIT1 binds to BRCA2/RAD51 complex and this binding is required for recruitment or retention of BRCA2/RAD51 complex at the DNA repair sites [39]. Thus, BRIT1 also functions directly in DNA repair via directing BRCA2/RAD51 foci to the DSBs. In consistent with the role of BRIT1 in regulating the DNA repair function of BRCA2/RAD51, the meiotic phenotypes in *BRIT1* ^−/−^ mice are virtually the same as those observed in mice with deficiency of BRCA2 [44] and DMC1 (a homologue of RAD51) [45],[46]. In these mice, spermatocytes are also arrested before or at the transition of zygotene to pachytene with aberrant chromosomal synapsis. In fact, like *BRIT1* ^−/−^ spermatocytes, *BRCA2* ^−/−^ spermatocytes also form DSBs without the consequent recruitment of RAD51 to the meiotic chromosome [44].

Our previous studies show that BRIT1 deficiency is correlated with genomic instability and breast cancer development [18]. Although our BRIT1 knockout mice within one and half years old did not develop any tumor, when we crossed BRIT1 knockout mice to the p53 null background, we found a significant effect of BRIT1 deficiency in enhancing cancer susceptibility (unpublished data). Notably, our very recent preliminary data indicate that low dosage of irradiation can readily induce breast tumors in the mice with conditional knockout of BRIT1 in the mammary gland but not in the control littermates. It will be very interesting to investigate if and to what extent BRIT1 deficiency may contribute to the initiation and progression of cancer with the existence of oncogenic or genotoxic stress. For example, we can assess whether crossing an activated Ras or HER2 allele into the BRIT1 deficient background will result in increased genomic instability and tumorigenesis compared to either mouse strain alone. Thus, our BRIT1 null mouse will be a very valuable model for further assessing BRIT1's role in genome maintenance and tumor suppression in the future.

## Materials and Methods

### Generation of *BRIT1* ^−/−^ mice and MEFs

We isolated *BRIT1* BAC clones from a 129/SvEv genomic library to construct the conditional targeting vector (Figure 1A). A 0.5 kb fragment consisting of exon 2 (E2), 3′ end of intron 1 and 5′ end of intron 2 was inserted between two *loxP* sites in the targeting vector, which allows to remove E2 after introduction of Cre in mice. A 3.0 kb fragment from intron 2 was cloned into the same vector as the 3′ homologous arm. A 5′ homologous arm (a 3.0 kb fragment containing E1 and 5′ of intron 1) was subsequently cloned into the vector. The Neo selection marker can be excised via the recombination of the Frt sites after introduction of Flp recombinase, which could avoid any unexpected effect of Neo cassette on the normal splicing of *BRIT1*. The targeting vector was linear zed by *Pac*I and electroporated into AB2.2 ES cells. Neomycin-resistant colonies were selected with Geneticin and analyzed for the expected homologous recombination by *EcoR*V digestion followed by Southern blot analysis using a 5′ flanking probe. The targeted clones were then confirmed using a 3′ flanking probe after *BamH*I digestion of the genomic DNA.

To generate the mice with *BRIT1* conditional targeting allele, two targeting ES cells were injected into C57BL/6J mouse blastocysts. The injection was carried out by the Engineered Mouse Core in department of Molecular and Human Genetics at Baylor College of Medicine [47]. Male chimeras with 95% agouti color were bred with C57BL/6J females and germ-line transmission of the *BRIT1* targeting allele was confirmed by agouti coat color in F1 animals and by Southern blot analysis on mouse tail DNA using the two flanking probes for *BRIT1*. These heterozygous mice were crossed with Flp mice to generate the mice carrying one *BRIT1* conditional allele with the excision of the Neo cassette (*BRIT1* ^+/co^). These mice were then bred with transgenic mice carrying a Cre gene under the control of β-actin promoter to generate *BRIT1* ^+/−^ mice. *BRIT1* ^+/−^ mice were further bred to generate *BRIT1* ^−/−^ mice.

Primary MEFs were obtained from embryonic days E14.5 by a standard procedure. MEFs were grown in DMEM supplemented with 10% FBS, 1% penicillin/streptomycin and 0.1% Fungazone and kept at a low passage for future use.

### Radiosensitivity of MEFs and whole mice

Three plates of *BRIT1* ^+/+^ or *BRIT1* ^−/−^ MEFs were exposed to IR (0, 2, 4, or 6 Gy), and survival rates were calculated 6 days after IR. Whole-body γ-irradiation of mice was carried out as described previously [48]. Ten pairs of *BRIT1* ^+/+^/*BRIT1* ^+/−^ and *BRIT1* ^−/−^ littermates were irradiated with 7 Gy of whole body IR, and survival rates were calculated every two days after irradiation.

### Metaphase spreads of lymphocytes and MEFs

Mice were sacrificed at postnatal day P42, and splenocytes were crushed though a mesh into a collection tube, and lymphocytes were separated using Lymph-M method (Accurate Chemical & Scientific Corp). The separated lymphocytes were activated with ConA and IL-2. MEFs were γ-irradiated with a dose of 1 Gy, and then collected for metaphase spread 3 hr or 20 hr post-IR. Cytological preparation of the activated T cells and γ-irradiated MEFs was made as described previously [49]. From each sample, at least 30–35 metaphase spreads were analyzed.

### Immunofluoresent staining of γ-irradiation induced foci formation

MEFs on coverslips were treated with or without IR, and underwent immunoflurorescent (IF) staining at the indicated time points as described previously [18]. The primary antibodies used here were anti-RAD51 (BD pharmingen) and anti-γ-H2AX (Bethyl). The coverslips were finally mounted onto glass slides with VectaShield antifade (Vector Laboratories) and visualized by using a Zeiss Axiovert 40 CFL fluorescence microscope.

### Histological and immunoflurescent staining of testes sections

Testes were obtained from *BRIT1* ^+/+^ and *BRIT1* ^−/−^ mice at different ages (P7, P14, P21, P28, and P56), fixed in 4% paraformaldehyde at 4°C, and routinely embedded in paraffin. Haematoxylin-eosin (H&E) staining was performed according to the standard procedure. Slides were de-paraffinized and rehydrated, and antigen retrieval was carried out by incubating the slides in 0.01 M sodium citrate buffer (pH 6.1) in a hot water bath at 95°C for 30 min. If necessary, endogenous peroxidase activity was quenched with 3% hydrogen peroxide in methanol for 10 min at room temperature. Primary antibodies used here are anti-SOX-9, anti-Tra98 (gifts from Drs. Pumin Zhang and Xingxu Huang, Baylor College of Medicine, Houston, TX). Sections were finally visualized using a Zeiss Axiovert 40 CFL microscope, and images were captured with a Zeiss AxioCam MRc5 digital camera.

### Surface-spread and immunofluoresent analyses

Seminiferous tubules were collected from 2- to 4-month-old mice, and tubule segments were isolated in 1×DPBS. Chromosome spread of spermatocytes was made following the protocol elsewhere [50],[51]. The following primary antibodies were used for immunofluorescent analyses: rabbit anti-SPO11 (Santa Cruz), rabbit anti-γ-H2AX (Bethyl), rabbit anti-RAD51 (Sigma), rabbit anti-SCP1 (GeneTex), rabbit anti-SCP3 (GeneTex) and guinea pig anti-SCP3 (gift from Dr. Ricardo Benavente, University of Würzburg, Würzburg, Germany). Alexa 488-coupled anti-rabbit IgG and/or Alexa 594-conjugated anti-guinea pig IgG were used as secondary antibodies. The slides were finally counterstained and mounted with antifade mounting medium with DAPI (Vector Laboratories). Foci were visualized microscopically with oil-immersed objectives, captured with a digital camera (Zeiss AxioCam MRc5), and processed with Photoshop (Adobe).

### Chromatin isolation

Chromatin isolation assay were performed as previously described [18]. Briefly, MEFs (totally 5×106 cells) were treated with or without IR (8 Gy), collected 1 h later, and then washed with PBS. The cells pellets were resuspended in 200 µl of solution A (10 mM HEPES [pH 7.9], 10 mM KCl, 1.5 mM MgCl_2_, 0.34 M sucrose, 10% glycerol, 1mM dithiothreitol, 10m MNaF, 1mM Na_2_VO_3_, and protease inhibitors). Triton X-100 was added to a final concentration of 0.1%, and the cells were incubated for 5 min on ice. Cytoplasmic proteins were separated from nuclei by low-speed centrifugation. The isolated nuclei (P1) were washed once with solution A and then lysed in 200 µl of solution B (3 mM ethylenediamine tetraacetic acid, 0.2 mM EGTA, 1 mM dithiothreitol, and protease inhibitors). Insoluble chromatin was collected by centrifugation, washed once in solution B, and centrifuged again for 1 min. The final chromatin pellets (P3) were digested by resuspending nuclei in solution A containing 1 mM CaCl_2_ and 50 units of micrococal nuclease and incubated at 37°C for 1 min, after which the nuclease was stopped by addition of 1 mM EGTA. The chromatin pellets (MNase-digested P3) were resuspended in 2× Laemmli buffer and boiled 10 min at 70°C. Following centrifugation at high speed (13,000 rpm), the chromatin associated proteins in the supernatant were analyzed by SDS-PAGE/Western blotting assay.

## Supporting Information

Figure S1 Smaller testes and more apoptotic spermatogenic cells in *BRIT1*-deficient mice. (A) *BRIT1*-deficient testes were much smaller than WT after postnatal days 21 (P21). (B–D) *BRIT1*-deficient spermatogenic cells were prone to be apoptotic. The apoptotic cells were determined with TUNEL assay. The representative apoptotic spermatogenic cells from WT and mutant testes at P28 were shown in (B). Although the percentage of apoptotic tubules between mutant and WT was only significantly different in P28 (C), the apoptotic cells were dramatically increased in *BRIT1*-deficient testes after P14 (D). Scale bar in B, 100 µm. * *P*<0.05 compared with the wild-type control.
(7.16 MB TIF)

Figure S2 *BRIT1*-deficient ovaries were smaller with no ovarian follicles. (A) *BRIT1*-deficient ovaries were much smaller than WT. The relative weight of ovaries from *BRIT1* ^+/+^ and *BRIT1* ^−/−^ mice at P35 were calculated. (B) There was no ovarian follicles in *BRIT1*-deficient ovaries. The WT and mutant ovary tissues were sectioned and stained with H&E. Here shown were the ovaries at postnatal days 8. Black arrows, the whole ovaries; blue arrows, primary follicles. Scale bar, 0.2 mm.
(4.88 MB TIF)

Figure S3 Expression pattern of BRIT1 in WT testicular tubules and foci formation in WT spermatocytes. (A) BRIT1 expressed in both spermagonia and spermatocytes of meiosis prophase I in WT. The WT-testes sections from indicated ages were stained using anti-BRIT1 antibody. BRIT1 was strongly expressed in spermagonia (blue arrow) and spermatocytes (black arrow). No BRIT1 was detected in *BRIT1*-deficient testes. Scale bar: 50 µm. (B) BRIT1 foci were abundantly formed on leptotene/zygotene chromosomes in WT spermatocytes. BRIT1 foci formation occurred at leptotene and peaked during zygotene. During pachytene when spermatocytes complete the synapsis, BRIT1 foci mainly localized at the non-synapsed sex body (white arrow) and the telomeres. Scale bar, 10 µm.
(6.92 MB TIF)

Figure S4 RAD51 protein expression was not altered in *BRIT1*-deficient testes. Total protein lysates from indicated testes were used to detect the protein levels of RAD51. RAD51 expression was comparable between the WT and mutant testes. Actin was detected as a loading control.
(0.29 MB TIF)

Table S1 Chromosomal aberrations increased in activated T cells from *BRIT1^−/−^* spleens.
(0.04 MB DOC)

## References

[1] Pastink A, Eeken JC, Lohman PH (2001). Genomic integrity and the repair of double-strand DNA breaks.. Mutat Res.

[2] Franco S, Alt FW, Manis JP (2006). Pathways that suppress programmed DNA breaks from progressing to chromosomal breaks and translocations.. DNA Repair.

[3] Pandita TK, Hittelman WN (1992). The contribution of DNA and chromosome repair deficiencies to the radiosensitivity of ataxia-telangiectasia.. Radiat Res.

[4] Kuzminov A (1995). Collapse and repair of replication forks in Escherichia coli.. Mol Microbiol.

[5] Richardson C, Horikoshi N, Pandita TK (2004). The role of the DNA double-strand break response network in meiosis.. DNA Repair.

[6] Rooney S, Chaudhuri J,  Alt. FW (2004). The role of the non-homologous end-joining pathway in lymphocyte development.. Immunol Rev.

[7] Zhou BB, Elledge SJ (2000). The DNA damage response: putting checkpoints in perspective.. Nature.

[8] Rouse J, Jackson SP (2002). Interfaces between the detection, signaling, and repair of DNA damage.. Science.

[9] Stucki M, Clapperton JA, Mohammad D, Yaffe MB, Smerdon SJ (2005). MDC1 directly binds phosphorylated histone H2AX to regulate cellular responses to DNA double-strand breaks.. Cell.

[10] Paull TT, Rogakou EP, Yamazaki V, Kirchgessner CU, Gellert M (2000). A critical role for histone H2AX in recruitment of repair factors to nuclear foci after DNA damage.. Curr Biol.

[11] Chapman JR, Jackson SP (2008). Phospho-dependent interactions between NBS1 and MDC1 mediate chromatin retention of the MRN complex at sites of DNA damage.. EMBO Rep.

[12] Schultz LB, Chehab NH, Malikzay A, Halazonetis TD (2000). p53 binding protein 1 (53BP1) is an early participant in the cellular response to DNA double-strand breaks.. J Cell Biol.

[13] Rappold I, Iwabuchi K, Date T, Chen J (2001). Tumor suppressor p53 binding protein 1 (53BP1) is involved in DNA damage-signaling pathways.. J Cell Biol.

[14] Sung P, Krejci L, Van Komen S, Sehorn MG (2003). Rad51 recombinase and recombination mediators.. J Biol Chem.

[15] Scully R, Chen J, Ochs RL, Keegan K, Hoekstra M (1997). Dynamic changes of BRCA1 subnuclear location and phosphorylation state are initiated by DNA damage.. Cell.

[16] Thorslund T, West SC (2007). BRCA2: a universal recombinase regulator.. Oncogene.

[17] Lin SY, Rai R, Li K, Xu ZX, Elledge SJ (2005). BRIT1/MCPH1 is a DNA damage responsive protein that regulates the Brca1-Chk1 pathway, implicating checkpoint dysfunction in microcephaly.. Proc Natl Acad Sci USA.

[18] Rai R, Dai H, Multani AS, Li K, Chin K (2006). BRIT1 regulates early DNA damage response, chromosomal integrity, and cancer.. Cancer Cell.

[19] Jackson AP, Eastwood H, Bell SM, Adu J, Toomes C (2002). Identification of microcephalin, a protein implicated in determining the size of the human brain.. Am J Hum Genet.

[20] Xu X, Lee J, Stern DF (2004). Microcephalin is a DNA damage response protein involved in regulation of CHK1 and BRCA1.. J Biol Chem.

[21] Alderton GK, Galbiati L, Griffith E, Surinya KH, Neitzel H (2006). Regulation of mitotic entry by microcephalin and its overlap with ATR signalling.. Nat Cell Biol.

[22] Sonoda E, Sasaki MS, Morrison C, Yamaguchi-Iwai Y, Takata M (1999). Sister chromatid exchanges are mediated by homologous recombination in vertebrate cells.. Mol Cell Biol.

[23] González-Barrera S, Cortés-Ledesma F, Wellinger RE, Aguilera A (2003). Equal sister chromatid exchange is a major mechanism of double-strand break repair in yeast.. Mol Cell.

[24] Wood JL, Liang Y, Li K, Chen J (2008). Microcephalin/MCPH1 associates with the Condensin II complex to function in homologous recombination repair.. J Biol Chem.

[25] Peng G, Yim EK, Dai H, Jackson AP, Burgt I (2009). BRIT1/MCPH1 links chromatin remodelling to DNA damage response.. Nat Cell Biol.

[26] Bellvé AR, Cavicchia JC, Millette CF, O'Brien DA, Bhatnagar YM (1977). Spermatogenic cells of the prepuberal mouse. Isolation and morphological characterization.. J Cell Biol.

[27] Zhao GQ, Garbers DL (2002). Male germ cell specification and differentiation.. Dev Cell.

[28] Dobson MJ, Pearlman RE, Karaiskakis A, Spyropoulos B, Moens PB (1994). Synaptonemal complex proteins: occurrence, epitope mapping and chromosome disjunction.. J Cell Sci.

[29] Schalk JA, Dietrich AJ, Vink AC, Offenberg HH, van Aalderen M (1998). Localization of SCP2 and SCP3 protein molecules within synaptonemal complexes of the rat.. Chromosoma.

[30] Dietrich AJ, Mulder RJ (1983). A light- and electron microscopic analysis of meiotic prophase in female mice.. Chromosoma.

[31] de Vries FA, de Boer E, van den Bosch BM, Baarends WM, Ooms M (2005). Mouse Sycp1 functions in synaptonemal complex assembly, meiotic recombination, and XY body formation.. Genes Dev.

[32] Keeney S, Giroux CN, Kleckner N (1997). Meiosis-specific DNA double-strand breaks are catalyzed by Spo11, a member of a widely conserved protein family.. Cell.

[33] Fernandez-Capetillo O, Lee A, Nussenzweig M, Nussenzweig A (2004). H2AX: the histone guardian of the genome.. DNA Repair.

[34] Kinner A, Wu W, Staudt C, Iliakis G (2008). Gamma-H2AX in recognition and signaling of DNA double-strand breaks in the context of chromatin.. Nucleic Acids Res.

[35] Keeney S (2001). Mechanism and control of meiotic recombination initiation.. Curr Top Dev Biol.

[36] Mahadevaiah SK, Turner JM, Baudat F, Rogakou EP, de Boer P (2001). Recombinational DNA double-strand breaks in mice precede synapsis.. Nat Genet.

[37] Ogawa T, Yu X, Shinohara A, Egelman EH (1993). Similarity of the yeast RAD51 filament to the bacterial RecA filament.. Science.

[38] Tarsounas M, Morita T, Pearlman RE, Moens PB (1999). RAD51 and DMC1 form mixed complexes associated with mouse meiotic chromosome cores and synaptonemal complexes.. J Cell Biol.

[39] Wu X, Mondal G, Wang X, Wu J, Yang L (2009). Microcephalin regulates BRCA2 and Rad51-associated DNA double-strand break repair.. Cancer Res.

[40] Xu Y, Ashley T, Brainerd EE, Bronson RT, Meyn MS (1996). Targeted disruption of ATM leads to growth retardation, chromosomal fragmentation during meiosis, immune defects, and thymic lymphoma.. Genes Dev.

[41] Celeste A, Petersen S, Romanienko PJ, Fernandez-Capetillo O, Chen HT (2002). Genomic instability in mice lacking histone H2AX.. Science.

[42] Lou Z, Minter-Dykhouse K, Franco S, Gostissa M, Rivera MA (2006). MDC1 maintains genomic stability by participating in the amplification of ATM-dependent DNA damage signals.. Mol Cell.

[43] Yang SZ, Lin FT, Lin WC (2008). MCPH1/BRIT1 cooperates with E2F1 in the activation of checkpoint, DNA repair and apoptosis.. EMBO Rep.

[44] Sharan SK, Pyle A, Coppola V, Babus J, Swaminathan S (2004). BRCA2 deficiency in mice leads to meiotic impairment and infertility.. Development.

[45] Pittman DL, Cobb J, Schimenti KJ, Wilson LA, Cooper DM (1998). Meiotic prophase arrest with failure of chromosome synapsis in mice deficient for Dmc1, a germline-specific RecA homolog.. Mol Cell.

[46] Yoshida K, Kondoh G, Matsuda Y, Habu T, Nishimune Y (1998). The mouse RecA-like gene Dmc1 is required for homologous chromosome synapsis during meiosis.. Mol Cell.

[47] Li K, Ramirez MA, Rose E, Beaudet AL (2002). A gene fusion method to screen for regulatory effects on gene expression: application to the LDL receptor.. Hum Mol Genet.

[48] Ward IM, Minn K, van Deursen J, Chen J (2003). p53 Binding protein 53BP1 is required for DNA damage responses and tumor suppression in mice.. Mol Cell Biol.

[49] Mei J, Huang X, Zhang P (2001). Securin is not required for cellular viability, but is required for normal growth of mouse embryonic fibroblasts.. Curr Biol.

[50] Peters AH, Plug AW, van Vugt MJ, de Boer P (1997). A drying-down technique for the spreading of mammalian meiocytes from the male and female germline.. Chromosome Res.

[51] Baart EB, de Rooij DG, Keegan KS, de Boer P (2000). Distribution of Atr protein in primary spermatocytes of a mouse chromosomal mutant: a comparison of preparation techniques.. Chromosoma.

