# Supplementary figures and images for: BRIT1/MCPH1 Is Essential for Mitotic and Meiotic Recombination DNA Repair and Maintaining Genomic Stability in Mice
Source: PLoS Genet. 2010 Jan 22;6(1):e1000826. doi: 10.1371/journal.pgen.1000826 (PMC2809772; doi:10.1371/journal.pgen.1000826)

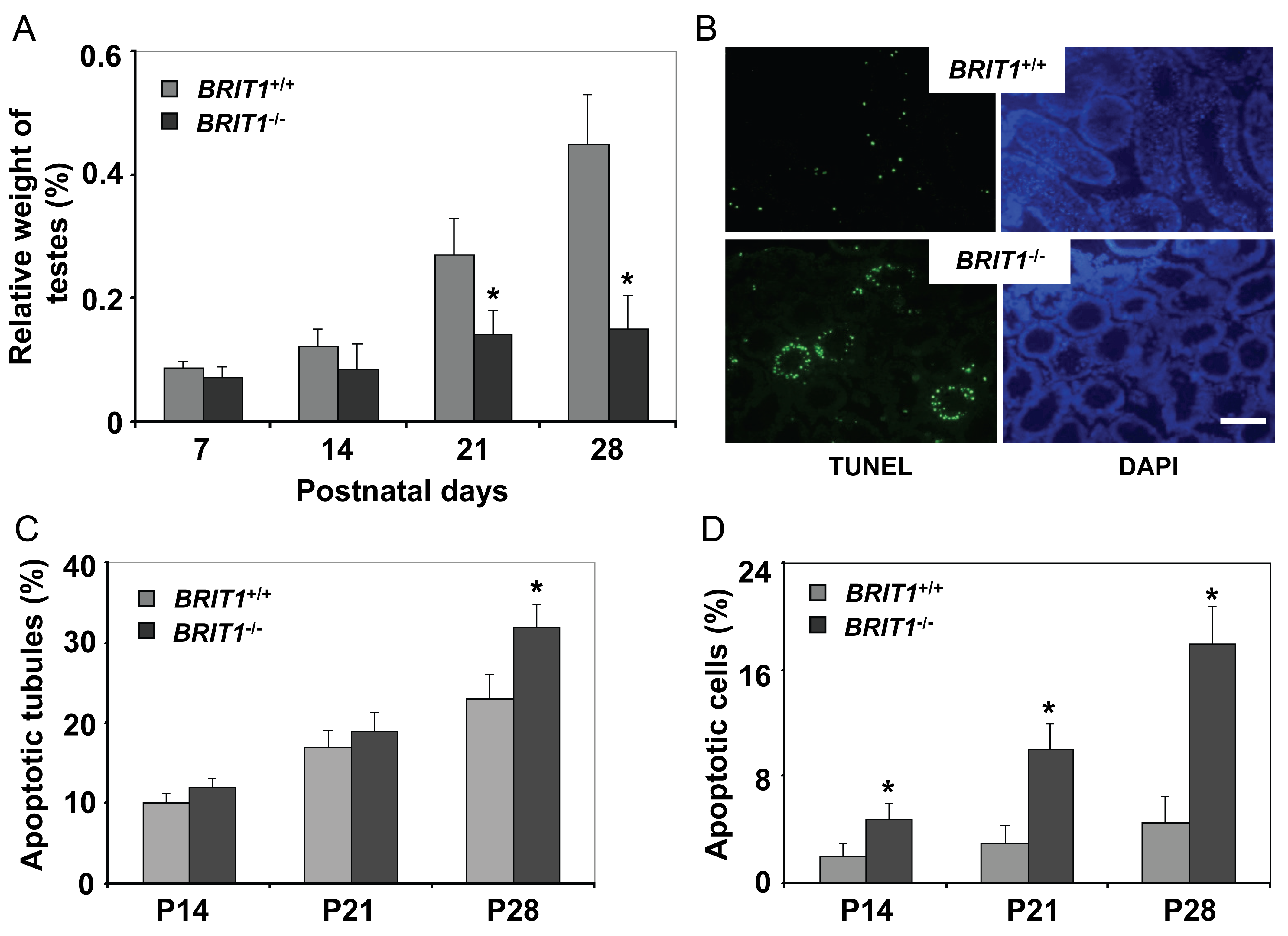

Supplement: Figure S1 — Smaller testes and more apoptotic spermatogenic cells in BRIT1-deficient mice. (A) BRIT1-deficient testes were much smaller than WT after postnatal days 21 (P21). (B–D) BRIT1-deficient spermatogenic cells were prone to be apoptotic. The apoptotic cells were determined with TUNEL assay. The representative apoptotic spermatogenic cells from WT and mutant testes at P28 were shown in (B). Although the percentage of apoptotic tubules between mutant and WT was only significantly different in P28 (C), the apoptotic cells were dramatically increased in BRIT1-deficient testes after P14 (D). Scale bar in B, 100 µm. * P<0.05 compared with the wild-type control. (7.16 MB TIF) [file pgen.1000826.s001.tif]

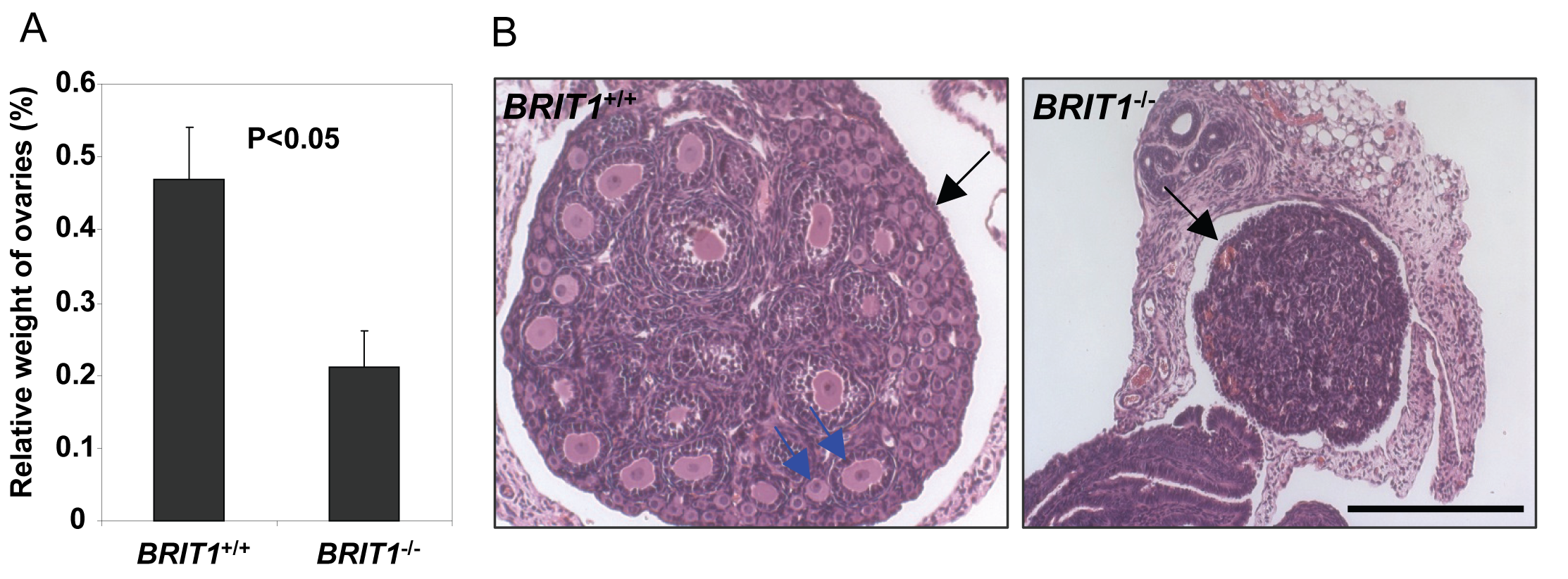

Supplement: Figure S2 — BRIT1-deficient ovaries were smaller with no ovarian follicles. (A) BRIT1-deficient ovaries were much smaller than WT. The relative weight of ovaries from BRIT1 +/+ and BRIT1 −/− mice at P35 were calculated. (B) There was no ovarian follicles in BRIT1-deficient ovaries. The WT and mutant ovary tissues were sectioned and stained with H&E. Here shown were the ovaries at postnatal days 8. Black arrows, the whole ovaries; blue arrows, primary follicles. Scale bar, 0.2 mm. (4.88 MB TIF) [file pgen.1000826.s002.tif]

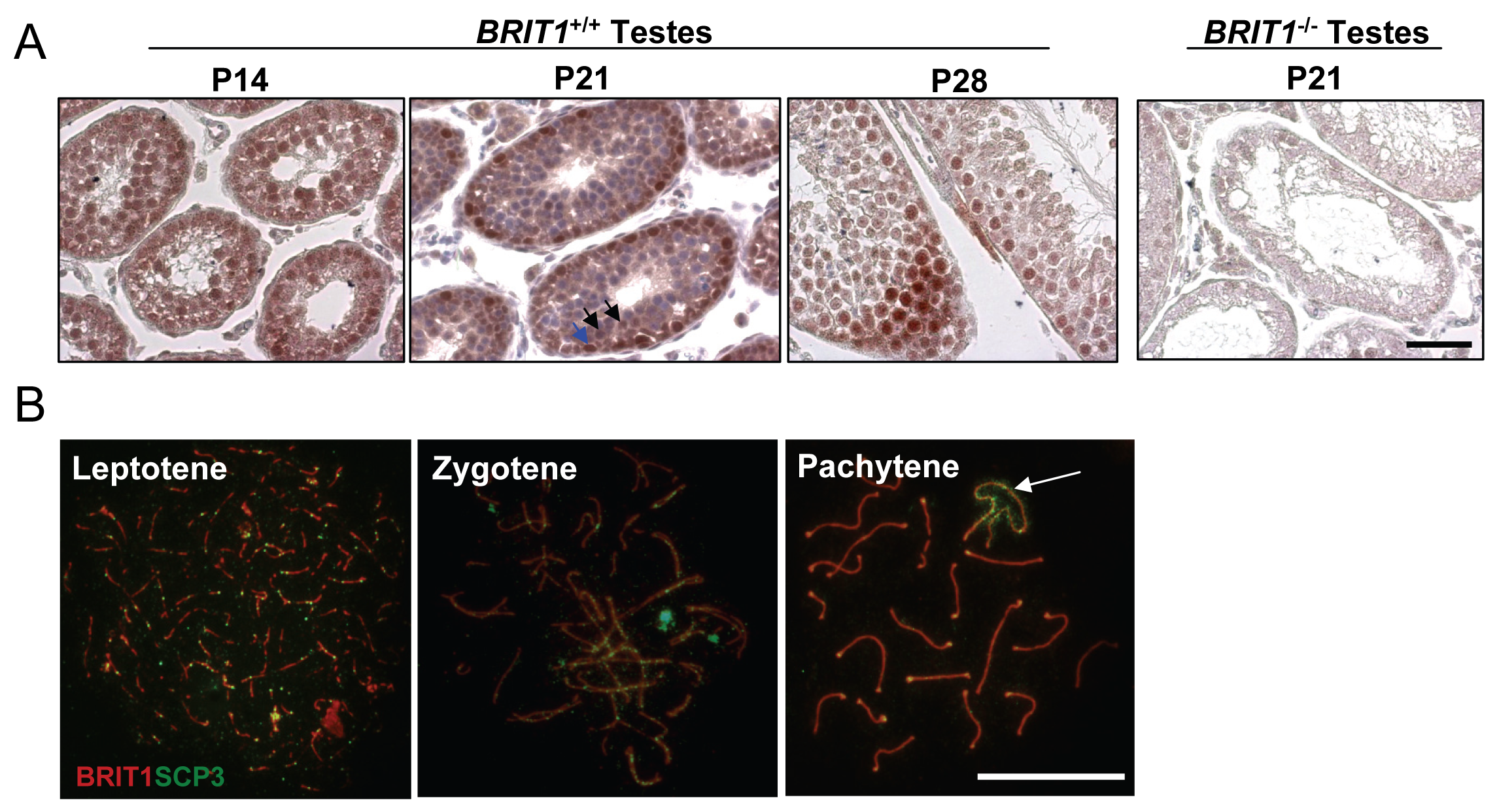

Supplement: Figure S3 — Expression pattern of BRIT1 in WT testicular tubules and foci formation in WT spermatocytes. (A) BRIT1 expressed in both spermagonia and spermatocytes of meiosis prophase I in WT. The WT-testes sections from indicated ages were stained using anti-BRIT1 antibody. BRIT1 was strongly expressed in spermagonia (blue arrow) and spermatocytes (black arrow). No BRIT1 was detected in BRIT1-deficient testes. Scale bar: 50 µm. (B) BRIT1 foci were abundantly formed on leptotene/zygotene chromosomes in WT spermatocytes. BRIT1 foci formation occurred at leptotene and peaked during zygotene. During pachytene when spermatocytes complete the synapsis, BRIT1 foci mainly localized at the non-synapsed sex body (white arrow) and the telomeres. Scale bar, 10 µm. (6.92 MB TIF) [file pgen.1000826.s003.tif]

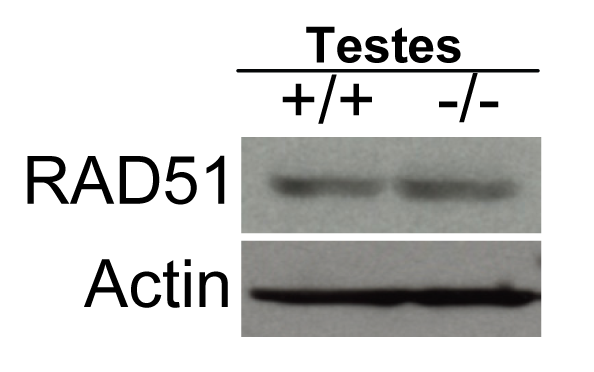

Supplement: Figure S4 — RAD51 protein expression was not altered in BRIT1-deficient testes. Total protein lysates from indicated testes were used to detect the protein levels of RAD51. RAD51 expression was comparable between the WT and mutant testes. Actin was detected as a loading control. (0.29 MB TIF) [file pgen.1000826.s004.tif]
