# Supplementary material for: BRIT1/MCPH1 Is Essential for Mitotic and Meiotic Recombination DNA Repair and Maintaining Genomic Stability in Mice
Source: PLoS Genet. 2010 Jan 22;6(1):e1000826. doi: 10.1371/journal.pgen.1000826 (PMC2809772; doi:10.1371/journal.pgen.1000826)
Supplement: Table S1 — Chromosomal aberrations increased in activated T cells from BRIT1−/− spleens. (0.04 MB DOC) [file pgen.1000826.s005.doc]

**Table S1**

| **Table S1: Chromosomal aberrations increased in activated T cells from *BRIT1*-/- spleens** | | | | |
| --- | --- | --- | --- | --- |
| Activated T cells | Number of  metaphase analyzed | Normal metaphases (%) | Aberrant metaphases (%) | Chromosomal breaks (%) |
| *BRIT1*^+/+^ | 35 | 91.4 | 8.6 | 8.6 |
| *BRIT1*^-/-^ (a) | 35 | 40 | 60 | 57.1 |
| *a*, 2.9% of total metaphases had chromosomal aberrations other than chromosomal breaks | | | | |
